# Facile synthesis of flexible macroporous polypropylene sponges for separation of oil and water

**DOI:** 10.1038/srep21265

**Published:** 2016-02-16

**Authors:** Guowei Wang, Hiroshi Uyama

**Affiliations:** 1 Department of Applied Chemistry, Graduate School of Engineering, Osaka University, Suita 565-0871, Japan

## Abstract

Oil spill disasters always occur accidentally, accompanied by the release of plenty of crude oil that could spread quickly over a wide area, creating enormous damage to the fragile marine ecological system. Therefore, the facile large-scale synthesis of hydrophobic three-dimensional (3-D) porous sorbents from low cost raw materials is in urgent demand. In this study, we report the facile template-free synthesis of polypropylene (PP) sponge by using a thermally-induced phase separation (TIPS) technique. The obtained sponge showed macroporous structure, excellent mechanical property, high hydrophobicity, and superoleophilicity. Oil could be separated from an oil/water mixture by simple immersing the sponge into the mixture and subsequent squeezing the sponge. All of these features make this sponge the most promising oil sorbent that will replace commercial non-woven PP fabrics.

Frequent oil spill accidents have been worldwide ecological, environmental, and economic issues threatening the human being. In 2010, the Gulf of Mexico oil spill released a record amount of crude oil and caused extensive damage to the natural ecosystem in the ocean or coastal waters. With the depletion of onshore crude oil, the exploration and production of offshore crude oil will become prominent in the future, increasing the risk of oil spill accidents.

*In situ* burning^1^,^2^,^3^, bioremediation^4^,^5^,^6^, dispersants^7^,^8^, dredging, skimming^9^, and solidifying are the commonly used techniques for the oil spill clean-up. However, these methods always cause adverse effects on the environment while often have poor efficiency. Hydrophobic three-dimensional (3-D) porous materials with high specific surface areas have strong ability to absorb oil or organic liquid from water phase, which is a facile approach for the treatment of oil spill^10^,^11^,^12^,^13^,^14^. Recently, numerous studies have focused on the design of the sorbents, *e.g.,* superhydrophobic melamine sponge^15^, marshmallow-like macroporous gel^16^, carbon nanofiber aerogel^17^,^18^,^19^,^20^, carbon nanotube sponge^21^, and spongy graphene^22^,^23^. All of those materials showed high hydrophobicity and compressibility, enabling the efficient absorption of oil and its recovery by squeezing the sorbents manually. Nevertheless, these methods are time-consuming for their preparation, and more importantly, need very expensive chemicals, thereby limiting their commercial applications. Oil spill always occur accidentally, and the spilled oil could spread over a wide area in a short time. Hence, it is challenging, but also indispensable, to develop an efficient, large-scalable, and economic method to produce oil sorbents from low cost raw materials.

Polypropylene (PP) is a commercially available hydrophobic-oleophilic polymer with methyl groups positioned at the backbone of the polymer chains. PP is chemically resistant to organic solvents, bases, and acids, which allows it to be used as oil sorbent without further modifications and crosslinkings. PP is reasonably economical, making it the ideal material for oil spill clean-up. Although nonwoven PP fabrics produced by electrospinning technique or melt-blown method have been widely used in oil spill clean-up^24^,^25^,^26^, they do not show compressible property, which prevents the usage of them for the recovery of absorbed oil through mechanical squeezing procedure. Furthermore, nonwoven PP fabrics face the problems such as high cost for device and long production time.

To solve the above problems, a spongy PP combining the compressible property of sponge and the low cost, hydrophobicity, and chemical resistance properties of PP should be a perfect sorbent for oil spill clean-up. With this inspiration, here we present the fabrication of a PP sponge using the thermally-induced phase separation (TIPS) technique^27^,^28^,^29^,^30^,^31^,^32^. The present PP sponge has many advantages over other hydrophobic 3-D porous materials: 1) All of the chemicals are commercially available and economical, making it possible to synthesize the sponge even in a commercial scale; and 2) No expensive devices are needed for the sponge fabrication, suggesting that the method is very suitable for industrial production.

## Results

### Fabrication of PP sponge

**Figure 1:**
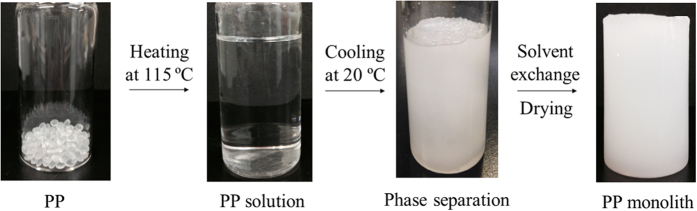
Fabrication procedure of PP sponge.

The PP sponge was fabricated by a TIPS method using the unique affinity of PP toward a mixed solvent of decalin and 1-butanol (Fig. 1). A PP solution was firstly prepared by dissolving PP in the solvent at 115 ^o^C. A PP sponge was obtained after cooling the solution at 20 ^o^C for 1 h. It should be noted that the phase separation completed in a short time, which is significant for the clean-up of emergency oil spill disasters. The PP sponge was subsequently immersed into acetone to remove the embedded molecule and dried under vacuum.

The effects of the solvent ratio and polymer concentration on the formation of PP sponge were studied. In the decalin ratio range from 40 to 60%, a compressible PP sponge in organic liquid (diethyl ether) was formed. Forty percent of decalin was determined as the best condition because the fabricated sponge showed better mechanical property. The polymer concentration also showed the large effect on the formation of PP sponge. A PP sponge with compressible property could be formed in the range of polymer concentration from 50 to 70 mg/mL. If the PP concentration was higher than 70 mg/mL, the obtained sponge was too hard to be compressed by hand. Below the concentration of 50 mg/mL, the sponge was too brittle to sustain large-scale compression. The lower concentration may result in insufficient numbers of polymer chains to form a tough PP sponge^33^.

**Figure 2:**
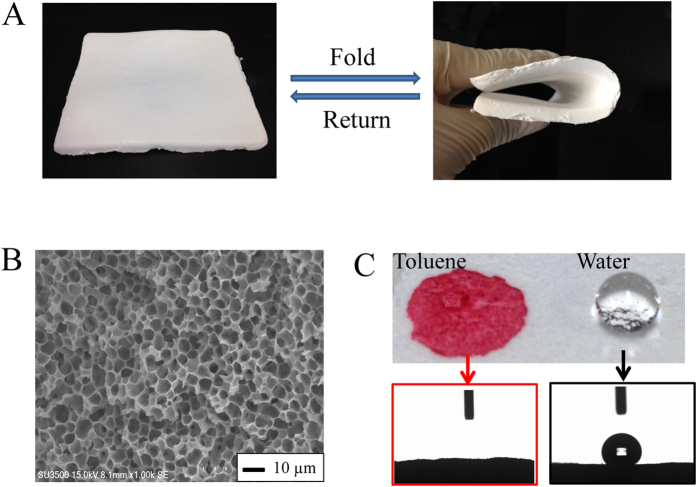
The flexibility of a large-size PP sponge (5 mm × 130 mm × 170 mm) (**A**), SEM image of the PP sponge (**B**), a toluene droplet (dyed with oil red) on the surface of the PP sponge with an oil contact angle of 0^o^ and a water droplet on the surface of the PP sponge with a water contact angle of 130^o^ (**C**).

The sponge had the ability to sustain large bending deformation and recover to its original shape without fracture (Fig. 2A), indicating its extraordinary flexibility over traditional carbon or silica-based oil sorbents. SEM image shows that the sponge had uniform interconnected macroporous structure with average pore size of ca. 5 μm (Fig. 2B). The macropores would act as cavities for the capture and storage of oil. The pore volume decreased by squeezing the sponge, inducing the recovery of the captured oil. The wetting property of the PP sponge surface was examined by measuring the contact angles. A water droplet stayed on the surface of the sponge displaying a water contact angle (CA_water_) of ca. 130^o^ (Fig. 2C). The rough surface of the PP sponge magnified by the water droplet could be clearly observed by naked eye, strongly indicating the hydrophobic surface of the sponge (Fig. 2C). On the contrary, once an oil droplet was deposited on the sponge surface, it was absorbed by the sponge rapidly (Fig. 2C). The high hydrophobic and superoleophilic (oil contact angle (CA_oil_): ca. 0^o^) properties are attributed to both the existence of many methyl groups along the backbone of the polymer chains, and the geometrical rough surface caused by the macroporous structure of the sponge. The sponge could float in water without sinking because of its light weight and hydrophobicity. At the same time, most of crude oils having lower densities than water tend to form a separated layer on the top of water. Thus, the present sponge is useful for oil spill clean-up.

### Mechanical property of PP sponge

**Figure 3:**
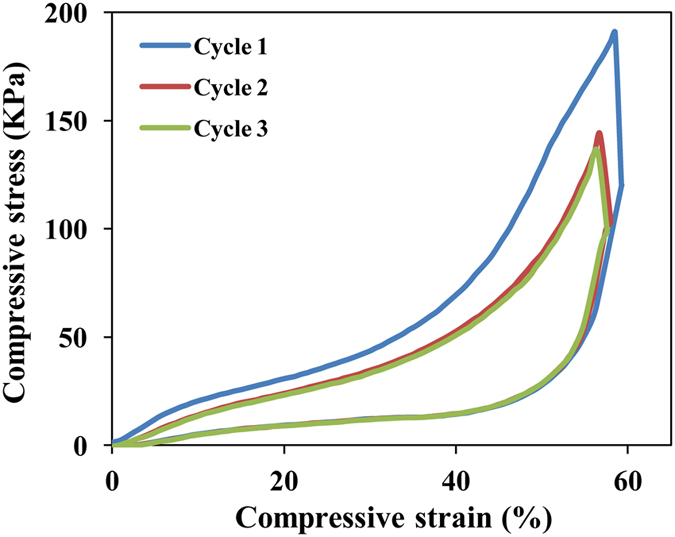
Cyclic stress-strain curves of the PP sponge at a maximum strain of 60%.

The PP sponge is not compressible in its dry state. However, it showed excellent compressibility in organic liquid, which is vital for the oil absorption application. As shown in Fig. 3, the sponge could bear a compression strain of 60% and recover to its original shape after unloading the compression stress. The area formed by the loading and unloading curves is the hysteresis loop which indicates the energy dissipated due to the internal friction of the sponge. The sponge showed large hysteresis loop, indicating large energy dissipation ability of the sponge. Cyclic compression test was measured to determine the creep property of the sponge. Compared with the first cycle, the second cycle showed a slightly decrease of compression stress, which is caused by the rearrangement of the long polymer chains. The third cycle showed similar the behaviour to the second cycle without significant decrease of compression stress. In all the cycles, the sponge recovered to its original state, which is significant for the application of oil recovery.

### Organic liquid/oil absorption

**Figure 4:**
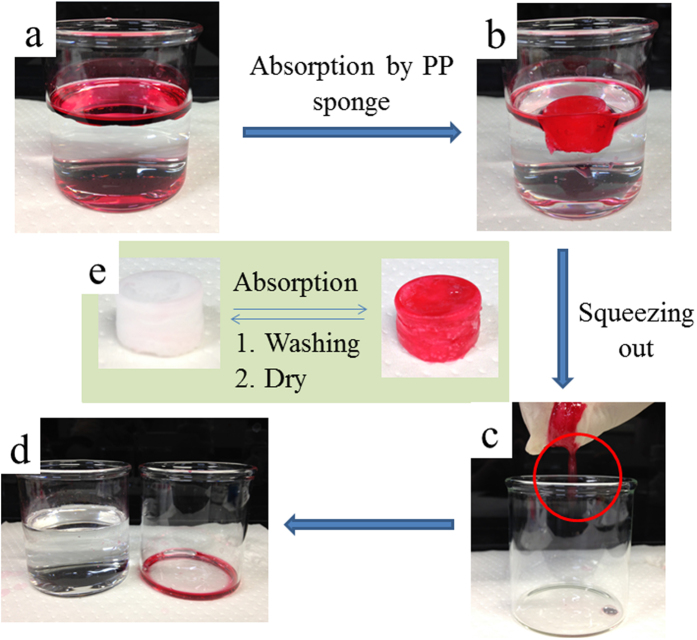
The absorption and recycling process of diethyl ether (dyed with oil red) and the recovery of the PP sponge by washing with volatile organic liquid (ethanol) and drying in air.

**Figure 5:**
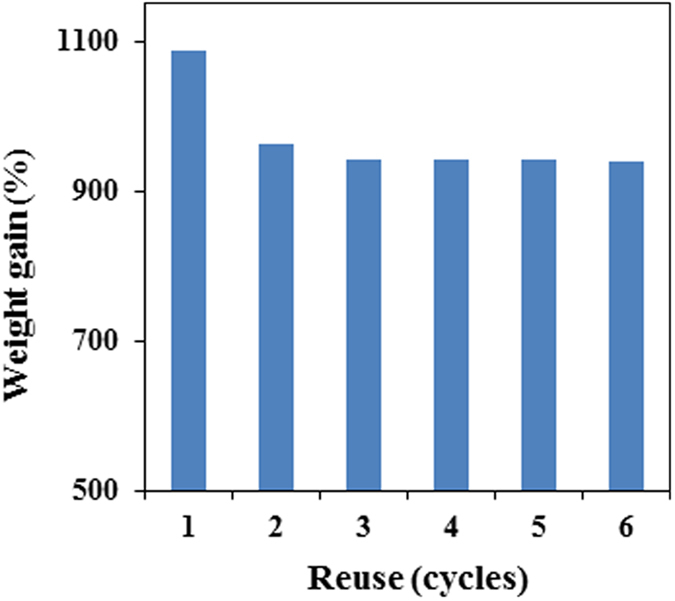
Absorption recyclability of the PP sponge toward diethyl ether.

As described above, the PP sponge is a very promising material for the separation of oil from water because of its 3-D interconnected macroporous structure, compressible property, high hydrophobicity, and superoleophilicity. To test this application, diethyl ether was selected as a model absorbate. A piece of PP sponge was placed in a mixture of diethyl ether (dyed with oil red) and water. After saturated absorption, the sponge still floated on the water and could be easily removed from the absorption system (Fig. 4b). The absorbed solvent, which is supposed to be stored mainly in the macrospores of the sponge, was recovered by simply squeezing the sponge manually (Fig. 4c). By repeating the absorption process several times, the organic liquid was separated from the water phase completely (supplementary movie S1). The porous structure and absorption ability of the sponge remained after many cycles of absorption/squeezing processes. Furthermore, the sponge could be reused by washing with volatile liquid and dried at room temperature (Figs 4e and 5).

**Figure 6:**
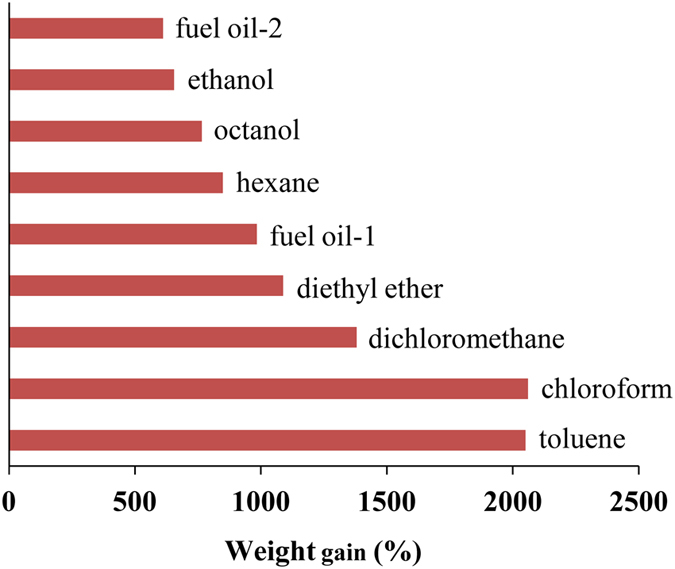
Absorption capacities of the PP sponge toward different organic liquids and oils.

To determine the absorption capability of the PP sponge, several common organic liquids and oils were selected for the absorption experiment. As shown in Fig. 6, the sponge absorbed up to 5–20 times of its own weight, higher than the commercially available PP fabric^15^. The PP sponge showed much higher absorption capability toward chloroform and toluene, which is caused by the swelling of PP sponge in these solvents. In other solvents (including viscous fuel oil-2), the PP sponge did not swell and maintained its shape and compressible property even after many absorption/squeezing cycles (supplementary movie S1). The excellent mechanical and absorption properties make the PP sponge a recyclable oil sorbent for large-scale oil spill clean-up.

## Discussion

We have developed a facile, large-scalable, and template-free method to fabricate a macroporous hydrophobic sponge from economical and commercially available PP using TIPS method. Phase separation completed in a short time, showing great advantage for the clean-up of a sudden oil spill disaster. The obtained sponge exhibited high flexibility, excellent mechanical property, high hydrophobicity, and superoleophilicity. In addition, the sponge showed good absorption ability toward several organic solvents/oils. The absorbed oils were easily recycled by squeezing the sponge manually. We believe that the present PP sponge is the most promising oil sorbent to replace commercial nonwoven PP fabrics.

## Methods

### Materials

Syndiotactic polypropylene with number average molecular weight of 7.5 × 10^4^ was purchased from Aldrich. Decalin and 1-butanol were supplied from Nacalai Tesque. Acetone and oil red were purchased from Wako Co. Two kinds of fuel oils (fuel oil-1 and fuel oil-2) with different kinematic viscosities were kindly supplied by JX Nippon Oil & Energy Corporation. The kinematic viscosities of fuel oil-1 and fuel oil-2 are below 2.0 × 10^−5^ m^2^/s, and between 5.0 × 10^−5^ m^2^/s and 2.5 × 10^−4^ m^2^/s, respectively (data by the supplier). All reagents were used as received without further purification.

### Fabrication of PP sponge

The PP sponge was generally prepared according to the following procedure (Fig. 1). A PP solution was prepared by dissolving 2.8 g of PP in a mixed solvent containing 16 mL of decalin and 24 mL of 1-butanol at 115 ^o^C. Afterwards, the solution was cooled at 20 ^o^C. During the cooling stage, the phase separation took place to form the PP sponge, which was immersed into a large amount of acetone to remove the embedded solvent and subsequently dried under vacuum.

### Oil absorption experiment

The absorption abilities of the PP sponge toward various organic solvents and oils were measured. The sponge was placed in a glass beaker filled with organic liquids or oils. After saturation, the sponge was taken out for weight measurements. The weights of the sponge before and after absorption were recorded for calculating the values of weight gain.

### Characterizations

The scanning electron microscope (SEM, Hitachi Co., SU3500) was used to observe the cross sections of PP sponge. All the samples were fractured to small pieces in liquid nitrogen and fixed on a SEM stub. Before observation, a thin layer of gold film was sputtered onto the surface of the samples. SEM images were recorded at an accelerating voltage of 15 kV. Contact angles of the sponge were measured with a Drop Master DM 300 (Kyowa Interface Science). Water droplet with a volume of 1.0 μL was fixed onto the sponge surface and the contact angle was determined at 2 s after the attachment of the droplet. The total porosity of the sponge was calculated as 88% by gravimetry using the equation described in the literature^34^. The density was measured as 114 kg/m^3^ by dividing the weight of one monolith with its volume.

Compressive test was performed by using a Haake Rheostress-6000 (Thermo Electron) with parallel plate geometry (diameter: 20 mm; gap: 1.0 mm). Before measurement, the sponge was immersed in diethyl ether for saturation absorption. It was cut to rectangular shape with the size of 1.0 cm × 1.0 cm × 1.5 cm. Afterwards, it was placed in a glass dish which was fixed on the stage. Diethyl ether was added to the glass dish until the diethyl ether level was higher than the top surface of the PP sponge. The strain ramp rate was set as 1.0 mm/min for all the tests.

## Additional Information

**How to cite this article**: Wang, G. and Uyama, H. Facile synthesis of flexible macroporous polypropylene sponges for separation of oil and water. *Sci. Rep.* **6**, 21265; doi: 10.1038/srep21265 (2016).

## Supplementary Material

Supplementary Information

Supplementary Movie S1
