# Supplementary material for: Facile synthesis of flexible macroporous polypropylene sponges for separation of oil and water
Source: Sci Rep. 2016 Feb 16;6:21265. doi: 10.1038/srep21265 (PMC4754692; doi:10.1038/srep21265)
Supplement: Supplementary Information [file srep21265-s1.pdf]

Facile synthesis of flexible macroporous polypropylene sponges for separation of oil and water

Guowei Wang, Hiroshi Uyama*

Department of Applied Chemistry, Graduate School of Engineering, Osaka University, Suita 565-0871, Japan

**E-mail: uyama@chem.eng.osaka-u.ac.jp*

Fax: +81-6-6879-7367; Tel: +81-6-6879-7364

Legend for supplemental movie:

This movie shows the oil-water separation process. A polypropylene sponge with the size of 15 mm × 7.5 mm × 7.5 mm was applied to absorb diethyl ether (dyed with oil red). The absorbed solvent, which is supposed to be stored mainly in the macrospores of the sponge, was recovered by simply squeezing the sponge manually. By repeating the absorption process several times, the organic liquid was separated from the water phase completely.
